# Galaxy HiCExplorer 3: a web server for reproducible Hi-C, capture Hi-C and single-cell Hi-C data analysis, quality control and visualization

**DOI:** 10.1093/nar/gkaa220

**Published:** 2020-04-17

**Authors:** Joachim Wolff, Leily Rabbani, Ralf Gilsbach, Gautier Richard, Thomas Manke, Rolf Backofen, Björn A Grüning

**Affiliations:** Bioinformatics Group, Department of Computer Science, University of Freiburg, Georges-Köhler-Allee 106, 79110 Freiburg, Germany; Max Planck Institute of Immunobiology and Epigenetics, Stübeweg 51, 79108 Freiburg im Breisgau, Germany; Institute for Cardiovascular Physiology, Goethe University, Frankfurt am Main, Germany; German Center of Cardiovascular Research (DZHK), Partner site RheinMain, Frankfurt am Main, Germany; Institute of Experimental and Clinical Pharmacology and Toxicology, Faculty of Medicine, University of Freiburg, Germany; INRAE, Agrocampus Ouest, Université de Rennes, IGEPP, F-35650 Le Rheu, France; Max Planck Institute of Immunobiology and Epigenetics, Stübeweg 51, 79108 Freiburg im Breisgau, Germany; Bioinformatics Group, Department of Computer Science, University of Freiburg, Georges-Köhler-Allee 106, 79110 Freiburg, Germany; Signalling Research Centres BIOSS and CIBSS, University of Freiburg, Schänzlestr. 18, 79104 Freiburg, Germany; Bioinformatics Group, Department of Computer Science, University of Freiburg, Georges-Köhler-Allee 106, 79110 Freiburg, Germany

## Abstract

The Galaxy HiCExplorer provides a web service at https://hicexplorer.usegalaxy.eu. It enables the integrative analysis of chromosome conformation by providing tools and computational resources to pre-process, analyse and visualize Hi-C, Capture Hi-C (cHi-C) and single-cell Hi-C (scHi-C) data. Since the last publication, Galaxy HiCExplorer has been expanded considerably with new tools to facilitate the analysis of cHi-C and to provide an in-depth analysis of Hi-C data. Moreover, it supports the analysis of scHi-C data by offering a broad range of tools. With the help of the standard graphical user interface of Galaxy, presented workflows, extensive documentation and tutorials, novices as well as Hi-C experts are supported in their Hi-C data analysis with Galaxy HiCExplorer.

## INTRODUCTION

Chromosome conformation capture (3C) (1) and its successors 4C (2,3), 5C (4) and Hi-C (5) have developed into the standard technologies used in studying the 3D conformation of chromatin. They can provide insights into the processes involved in chromatin folding and gene regulation. Hi-C technology is a well established method to study genome wide interaction of data and can detect large-scale chromosome structures, such as active and inactive (A/B) compartments (5,6), topological associated domains (TADs) (7,8), chromatin loop structures (9) or ratios of short to long range interaction counts. Although Hi-C is a powerful approach for studying the 3D structure of chromatin globally, it is limited in its ability to investigate location specific interactions, such as promoter-enhancer interactions, due to the need for high coverage and sequencing costs. Moreover, Hi-C is unable to capture protein-DNA interactions in the chromatin conformation context. To overcome these shortcomings, capture Hi-C (cHi-C) techniques have been developed. These assays are generating data, which are enriched for the predefined targets, such as promoter regions (Promoter cHi-C) (10), proteins or protein modifications (HiChIP) (11); HiChIP is able to capture chimeric protein-DNA interactions, including transcription factors or histone modifications. The location specific enrichment provides a significantly better signal-to-noise ratio and can therefore be used for a more location sensitive analysis. Capture Hi-C data cannot be analysed with established Hi-C algorithms and need their own tools. With the rise of single-cell sequencing technologies, the single-cell Hi-C (scHi-C) approach has been developed to allow for a deeper insight into the chromatin conformation dynamics between cell types, for instance during the cell cycle (12). For a review on the abilities and current developments of Hi-C and related techniques, the reviews of McCord *et al.* (13), Kempfer and Pombo (14) or Bonev and Cavalli (15) are recommended. The scHi-C analyses are much more resource intensive than Hi-C analyses and need specialized algorithms for dimension reduction. Galaxy HiCExplorer meets these requirements by providing efficient and easy to use tools for the analysis of Hi-C, cHi-C and scHi-C through a comprehensive and unified web server accessible at https://hicexplorer.usegalaxy.eu. It provides computational capabilities for even the most demanding analyses. Additionally, Galaxy HiCExplorer is easy to deploy locally thanks to the installer for a local Galaxy instance. Moreover, a command line version is provided by conda and is available via the bioconda channel (16).

## RELATED WORKS

Galaxy HiCExplorer is designed as an easy-to-use online service which is accessible through a web browser. Thus, no installation is required. By embedding it into Galaxy (17) and the https://usegalaxy.eu environment, it facilitates reproducible, shareable research as well as easily accessible data analysis. With Galaxy HiCExplorer, researchers can focus on their data analysis without facing any computational limitation or software dependency issue. To offer more flexibility, it is also possible to install Galaxy HiCExplorer on a local Galaxy instance. Hi-C data processing and downstream analysis are supported by many tool suites, such as Juicer (18), HiCUP (19), HOMER (20), HiC-Pro (21), HiFive (22) and the recently published HiCeekR (23). Juicer, HiC-Pro and HiCeekR offer several tools but are limited to a local installation. HiFive offers a Galaxy integration, but lacks the support of external data formats like *cool* file format (24). HiCUP and HOMER support only certain parts of Hi-C data analysis. Among the above tools, HiC-Pro is the only one with the ability to analyse cHi-C and HiChIP data. scHiCNorm (25) and scHiCluster(26) provide support for single-cell Hi-C data normalization and clustering, but suffer from the lack of a tool suite to guide researchers through the workflow of processing single-cell Hi-C data from the raw FASTQ files to the clustering of cells, including methods for building interaction matrices, quality control, dimension reduction and visualization. scHiCNorm and scHiCluster use text files to store the scHi-C interaction matrices, which are particularly space consuming, not easily shareable and prone to error accumulation. Galaxy HiCExplorer addresses all these shortcomings by providing a tool suite to support the analysis of Hi-C, captured Hi-C (e.g. Promoter cHi-C, HiChIP) and single-cell Hi-C data from the raw input data to publication ready results, as shown in Figure 2. Most importantly, none of the mentioned tools provide large computational resources to support Hi-C, cHi-C and single-cell Hi-C data analysis.

## GALAXY HICEXPLORER

Galaxy HiCExplorer offers a large collection of tools to pre-process, analyse and visualize Hi-C, cHi-C and scHi-C data. In addition to its assay-specific modules, users can benefit from the external pre-processing software for quality control of raw data and mappers such as BWA-MEM or Bowtie2 which are provided on the https://hicexplorer.usegalaxy.eu web server as well as the computational resources available. Moreover, for interactive Hi-C matrix exploration we have recently integrated HiGlass (27) into Galaxy. In the following, we briefly describe the new modules which have been added since our original publications on HiCExplorer 1 (28) and 2 (29).

### HiCExplorer

**Figure 1.**
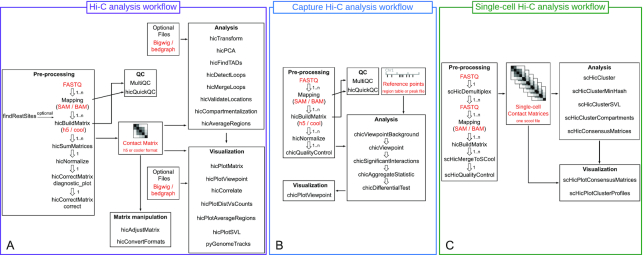
Analysis workflow for Hi-C (**A**), cHi-C (**B**) and scHi-C (**C**). All the workflows use the *hicBuildMatrix* to create the individual contact matrices. Hi-C and cHi-C supports HiCExplorer’s h5 and cool interaction matrix file format; however, scHi-C pipeline creates one cool file per cell. These files can then be merged into a single multi-cool (scool) matrix with *scHicMergeToSCool*.

HiCExplorer provides a variety of tools for a complete Hi-C data analysis, starting with tools to control the quality of data to create, adjust, normalize and correct interaction matrices. Furthermore, it provides tools for downstream analysis of Hi-C data such as identification of A/B compartments, TADs, loops or the computation of short versus long range contact ratios per chromosome. Finally, HiCExplorer has many options available for data visualisation such as plotting the interaction matrices, visualization of the detected TADs with pyGenomeTracks or creating aggregated contacts images. The workflow of Hi-C data analysis with Galaxy HiCExplorer is shown in Figure 1A. MultiQC, as shown in Figure 1A, supports HiCExplorer. If the structure of the quality report is changed, an update for MultiQC is necessary and the non-updated MultiQC might not work with the most recent quality report version.

#### Pre-processing

##### hicQuickQC

The creation of Hi-C interaction matrices, as well as the investigation of the quality of the data afterwards, may require a long processing time and is also resource intensive. To get a swift insight into the quality of Hi-C data, hicQuickQC has been introduced. It computes a quick summary of the Hi-C data quality using only a small subset of reads. The computation time to create the quality report with hicQuickQC for the first 1 million reads takes <3 min. The quality report is equal to the quality report of hicBuildMatrix and the only difference is that it is based only on the first 1 million reads instead of the full dataset.

##### hicFindRestSites

Hi-C interaction matrices with fixed size bins are not always the best representation of the data. In fact, with a sufficient sequencing depth, bins of a restriction fragment size are a better alternative. To generate such matrices, this tool generates a list of restriction sites for user-defined enzymes. This list can be used as an input to hicBuildMatrix to create restriction site resolution Hi-C matrices.

##### hicConvertFormat

Support for external interaction matrix data formats is missing in most Hi-C data analysis software. This makes it difficult to compare matrices which have been built with different software and to directly use them for further analysis. Instead, the matrices need to be built from scratch, which is time consuming and potentially error prone. This tool supports loading matrices of *cool*, HiCExplorers *h5*, Juicers *hic*, *Homer* and *HiCPro* format and can convert them to *cool*, *h5*, *Homer* and *ginteractions* (30) format.

##### hicNormalize

Normalization is a crucial step to be able to compare the interaction matrices obtained with a different sequencing depth. For this purpose, hicNormalize supports three normalization methods: (a) to the depth of the matrix with the least read coverage, (b) to the value range of 0 to 1 and (c) to a user defined scaling factor. For details on the normalization methods consult our Supplementary materials.

##### hicCorrectMatrix

Correcting the Hi-C interaction matrices is a necessary step to remove technical biases. In addition to the iterative correction (ICE) algorithm from Imakaev (31), HiCExplorer also offers the Knight-Ruiz correction (32), first used for Hi-C matrices by (9). The method is more memory efficient, is faster than the ICE algorithm and better suited for the analysis of high-resolution and deep read coverage interaction matrices.

#### Analysis

##### hicDetectLoops

**Figure 2.**
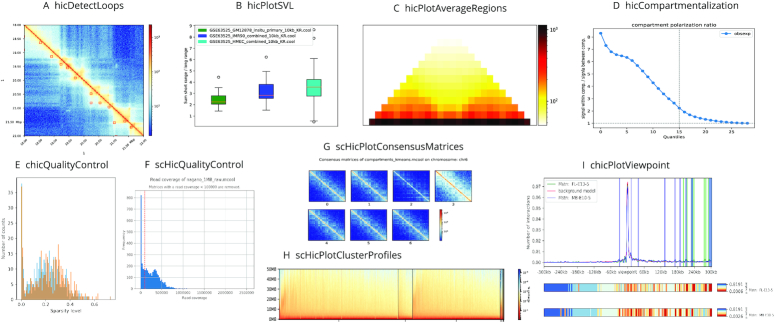
(**A**) Detected loops on *GM12878 primary* data from (9), computed by *hicDetectLoops* and visualised by *hicPlotMatrix*. (**B**) Short to long range contact interaction ratios created by *hicPlotSVL* on *GM12878 primary*, *IMR90* and *HMEC* data from (9). (**C**) Average regions of detected TADs from *hicFindTADs* on *GM12878 primary*, chromosome 1; data from (9). (**D**) The level of compartments separation on *GM12878 primary* data from (9), computed by *hicCompartmentalization*. (**E**) Quality control plot for *FL-E13-5* and *MB-E10-5* showing the sparsity distribution, data from (42). (**F**) Quality control plot for single-cell Hi-C data by (36). It shows the read coverage per cell, cells with <100 000 reads are discarded. (**G**) Consensus matrix plot for single-cell Hi-C data on 1 Mb resolution. Cells are dimension reduced by computing A/B compartments per cell and clustered with k-means. The consensus matrix of a cluster is the average of all interaction matrices of the cluster members. Data from (36). (**H**) Single-cell Hi-C cluster profile, created after dimension reduction by *scHicClusterMinHash* and spectral clustering on 1 Mb single-cell Hi-C data from (36). (**I**) Viewpoint of the gene *MSTN* on *FL-E13-5* and *MB-E10-5* with mean background and p-values per relative distance via continuous negative binomial distributions, data from (42).

Chromatin loops are long range chromatin interactions and present in Hi-C matrices as enriched regions in comparison to their local neighborhood. Depending on the read coverage and the resolution of the Hi-C interaction matrix, it is for instance possible to detect enhancer–promoter interactions. Due to its sensitivity to the read coverage it is recommended to run the loop detection on different resolutions and to merge them afterwards, using *hicMergeLoops*, into one loop file. By merging, overlapping loops are pooled into one loop. In addition, the tool *hicValidateLocations* can be used to confirm that the detected loops are correlated with detected locations of a protein of interest. For example, CTCF is known as a loop binding factor in mammals (7,9) and should therefore be present at many loop locations. Finally, the detected loops can be visualised with *hicPlotMatrix*, see Figure 2A. For details regarding the algorithm and benchmarks, consider (33).

##### hicCompartmentalization

This tool supports the analysis of interactions at the level of (active and inactive) compartments. These two large chromosomal domains can be defined through a principal component analysis (5) and are provided in Galaxy HiCExplorer by the existing *hicPCA* module. To visualize the difference in the interaction frequencies within and between the different compartments, a polarization plot can be generated using a method which was first introduced by (6). See Figure 2D.

##### hicAverageRegions

The comparison of specific regions between different samples can pose a challenge. One typical use case could be the comparison between multiple detected TADs on a wild type and a treatment sample. This tool extracts Hi-C submatrices corresponding to the upstream and downstream regions of reference anchors (e.g. a subset of TAD boundaries, promoter regions or any predefined positions of interest). It computes the average contacts of these submatrices and uses them to detect the potential differences of contact patterns located around these anchors, see Supplementary materials. The average of collected submatrices can be visualized with *hicPlotAverageRegions*, as shown in Figure 2C.

##### hicPlotSVL

Comparing the ratio of short range interaction to long range interaction between Hi-C matrices obtained in various experimental conditions can guide the understanding of chromatin topology and its folding principles. To this end, this tool computes the ratio per chromosome and plots it per sample as a boxplot, as shown in Figure 2B. For the mathematical details, please consult our Supplementary material.

##### pyGenomeTracks

The visualization tool *hicPlotTADs* which, was mentioned in the previous publication (29), came to the attention of many of our users. However, there was always some confusion as to whether or not it is for Hi-C data only which was never the case. To solve this, hicPlotTADs was renamed to pyGenomeTracks and is independently developed.

### Capture Hi-C

The cHi-C modules of HiCExplorer are designed for analysing Promoter cHi-C and HiChIP. HiCExplorer will also accept data from other Capture Hi-C methods, including ChiA-PET (34). if dedicated preprocessing steps were performed to obtain compatible mapping data. Furthermore, it can be used to generate virtual 4C plots from Hi-C data. As for Hi-C data, cHi-C interaction matrices are built with hicBuildMatrix. The regions of interest in these protocols, such as the location of the promoters in cHi-C or the binding sites of the target protein for HiChIP, are referred to as *reference points*. In the case of HiChIP, reference points are either annotated with peak calling tools, such as MACS2 (35) using either the HiChIP mapping file or ChIP-seq data, or regions (e.g. promoters) are manually selected. The region defined up- and downstream of a reference point is referred to as a *viewpoint*. Figure 2I illustrates all the up- and downstream distances within a viewpoint by their *relative distance* to a specific reference point. A background model is created which takes interactions per relative distance from all viewpoints into account. It is in the downstream analysis used to detect higher interactions as expected for a relative distance. These interactions are potentially different between a treatment and a control sample and therefore can be used for a differential test. The cHi-C workflow of Galaxy HiCExplorer is shown in Figure 1B. Please consult our Supplementary material concerning details of the presented cHi-C methods.

#### Pre-processing

##### chicQualityControl

This module is designed to investigate the quality of every single viewpoint, taking the sparsity of the interaction counts into account. A viewpoint will be removed if the sparsity of the data at this viewpoint is lower than a given threshold. To help users in setting an appropriate threshold, the tool generates several quality plots from which one is presented in Figure 2E.

##### chicViewpointBackground

The background model per relative distance is computed by taking all interaction counts of a relative distance over all viewpoints and samples into account. Based on this model, interactions with higher counts than an expected count will be identified during the downstream analysis.

#### Analysis

##### chicViewpoint

This tool extracts the interaction counts of each viewpoint from the interaction matrix, associates additional information and writes the viewpoint data to a file. Based on the background model, a *P*-value for each interaction count is computed. The *P*-value is an indicator if a specific count at a relative distance is in an expected range or higher.

##### chicSignificantInteractions

Using the *P*-values of a viewpoint, this tool decides via a threshold if an interaction at a relative distance is significant.

##### chicAggregateStatistic

The differential testing investigates if solitary interactions of two viewpoints have a different interaction frequency. These solitary interactions are either provided by a predefined target file or detected with *chicSignificantInteractions*. This tool aggregates the provided interactions from two viewpoints and prepares them as input for *chicDifferentialTest*.

##### chicDifferentialTest

The differential testing examines one solitary interaction between two viewpoints, under consideration of the interaction frequency at the reference points. As a differential test either chi^2^-test or Fisher’s test can be used under the null hypothesis that the interaction frequency is equal.

#### Visualization

##### chicPlotViewpoint

To visualize one or several viewpoints, *chicPlotViewpoint* has been introduced with the possibility of adding a mean background signal and highlighting the significant or differential interactions. Moreover, the computed p-values can be added as an additional heatmap as seen as in Figure 2I.

### Single-cell Hi-C

Single-cell Hi-C explores how chromatin is being folded and which elements contribute to its regulation on a single-cell scale. While analyzing Hi-C data is computationally expensive, this can increase drastically for scHi-C data. The reason for this is the increase in the number of Hi-C interaction matrices that need to be analysed from one to several thousand, with a corresponding increase in runtime and memory. The read coverage of scHi-C data is currently not high (36) and 1 megabase (Mb) resolution matrices are used to avoid generating highly sparse matrices. However, as sequencing costs decline, resolutions of 10 kb may be achievable and the demand for dimension reduction techniques, such as those presented here, will be indispensable. With scHiCExplorer, a software suite is provided to process single-cell Hi-C data offering tools for demultiplexing, matrix handling, correction, dimension reduction, clustering and visualisation. Figure 1C shows the workflow of single-cell Hi-C data analysis with Galaxy HiCExplorer. scHiCExplorer can be used for general processing of single-cell Hi-C data as long as the forward and reverse strand for each cell are provided as a BAM/SAM file. All pre-processing steps like adapter and/or barcode trimming, demultiplexing and mapping needs to be applied by third-party tools.

#### Pre-processing

##### scHicDemultiplex

Raw FASTQ data from a single-cell experiment usually contains reads from multiple cells which are encoded with different barcodes. This tool supports demultiplexing of an interleaved FASTQ file into one FASTQ file per cell. The demultiplexing is implemented to support the method which has been introduced in Nagano (36) for barcoding. Due to the lack of a standard method on how to encode barcodes, presently, demultiplexing is limited to FASTQ files with the same barcoding method as in (36). Other demultiplexing tools are part of the general Galaxy tool suite.

##### scHicMergeToSCool

Every single-cell interaction matrix can be created with *hicBuildMatrix*. *scHicMergeToSCool* can merge individual matrices into a joint matrix in multicool format (24), which will be used in all subsequent downstream analysis and visualization tools. While using the API of cooler, the data is not stored with multiple resolutions as it is defined by (24). The cool file is used as a container format for the individual cool files of the Hi-C matrices. For this reason, the format is referred to as *scool*.

##### scHicQualityControl

Since scHi-C data is a very sparse, not all matrices have sufficient read coverage to be considered for the downstream analysis. Thus, the quality control module removes interaction matrices of cells with total read counts below a user-specified threshold (see Figure 2E) or very sparse interaction matrices.

##### scHicCreateBulkMatrix

This tool supports to pool all matrices stored in the *scool* file to one single Hi-C interaction matrix and enables the analysis like in regular Hi-C.

Several modules of HiCExplorer are also required in single-cell Hi-C data analysis. To provide an equal functionality at the single cell level and to support the scool file format, scHiCExplorer reuses these modules from HiCExplorer. These are *scHicNormalize*, *scHicCorrectMatrices*, *scHicAdjustMatrix*, *scHicMergeMatrixBins* and *scHicInfo*. scHiCExplorer adds the functionality of handling the multiple matrices stored in the scool file and distributes the computations over several threads.

#### Dimension and clustering reduction

Clustering cells is a common approach to study the difference between them and to learn about their relations from single cell data. scHiCExplorer provides the *k-means* and *spectral* clustering methods. K-means was used on scHi-C data by (36) or (26), but the choice of a clustering algorithm is always dependent on the data. For this reason, scHiCExplorer provides additional the spectral clustering and will continue adding standard clustering algorithms in the future. However, reducing the dimensions of the underlying matrices is necessary to be able to cluster cells in a reasonable amount of time and to decrease the memory footprint; as shown in Supplementary Table S1. The usage of dimension reduction is also often necessary to achieve good results (37–39). The results in the Supplementary Figures S1–S4 confirm this. The need to reduce the dimensions becomes obvious when matrices of higher resolutions are used. The combined raw data matrix for a scHi-C dataset has a dimensionality of cells*features, where *features* = bins*bins for one matrix. As an example, mapping of the Nagano 2017 (36) data on the mouse mm9 genome and using it to make a 1 Mb resolution matrix, will already return a matrix of 2500*7.3 million dimensions; this number will increase to 2500*7.3 billion dimensions if the resolution of the matrix increases to 10 kb.

##### scHicCluster

A principal component analysis (reducing to samples*bins) or a *k*-nearest neighbors matrix (reducing to samples*samples) can be chosen as the desired method to reduce the dimensions of data. However, a clustering of the raw data without applying any dimension reduction is also supported.

##### scHicClusterMinHash

Clustering and dimension reduction techniques of *scHicCluster* usually work with low resolutions like 1 Mb but require a large amount of memory (>1 TB) on matrices of higher resolutions such as 10 kb. MinHash (40) is an approximate nearest neighbors method which computes the *k*-nearest neighbors matrix via local sensitive hash functions and reduces the number of dimensions to samples*samples. MinHash’s approximate computation of the *k*-nearest neighbors makes it possible to process 10 kb resolution scHi-C data. Our implementation runs for just over one hour and needs 53GB of memory, for more details consider (41).

##### scHicClusterSVL

This dimension reduction method computes the ratio of short range and long range contacts per chromosome and reduces the dimensions of the matrix to samples*chromosomes.

##### scHicClusterCompartments

This method computes the A/B compartments of each cell and clusters cells based on their compartments. It reduces the matrix dimensions to samples*bins.

#### Visualization

Due to the high dimensionality of matrices per cell ( bins*bins ), a satisfactory visual representation of single-cell Hi-C data clustering is difficult to achieve. Traditional methods represent the data in a two dimensional space; however, decreasing dimensionality from a few million (e.g. a 1 Mb resolution matrix) or billion (e.g. a 10 kb resolution matrix) to two dimensions will create a non-meaningful representation. scHiCExplorer offers two alternative representations of cells’ clusters: Per cluster (a) a consensus matrix of all cells is plotted or (b) each cell of a cluster is visualized with its decreasing contact frequency by increasing the distance from the main diagonal.

##### scHicConsensusMatrices

Using the results of the clustering, this tool merges all matrices of one cluster into a single interaction matrix and normalizes the resulting consensus matrices to the same read coverage. This matrix can be visualized as the consensus matrix of a cluster by *scHicPlotConsensusMatrices* and reveals the clustering power in separation of the cells based on their chromatin density. See Figure 2G.

##### scHicPlotClusterProfiles

A cluster profile shows the decrease of contact frequencies per cell from the main diagonal to 50 Mb distance from it. A good clustering is achieved if the decreasing of contact frequency is similar for all cells of a cluster and if the profiles of various clusters differ. Figure 2H shows the different cells grouped by clusters on the x-axis and the decreasing contact frequency by an increasing distance from the main diagonal on the y-axis.

## IMPLEMENTATION

Galaxy HiCExplorer is implemented as a collection of Galaxy tool wrappers and is available on the Galaxy ToolShed. The Galaxy integration is provided for HiCExplorer as well as scHiCExplorer. HiCExplorer and scHiCExplorer are both implemented in Python 3.6 and are available on Bioconda (16). The Knight-Ruiz correction and the MinHash approximate *k*-nearest neighbors for the dimension reduction are implemented in C++ and are also available on Bioconda.

## USING HICEXPLORER

### Installation and usage

Galaxy HiCExplorer can be used as a web server and is accessible via https://hicexplorer.usegalaxy.eu. All presented tools are publicly available and may be used without any required registration. Unregistered users are provided with 11 GB storage space, while registered users are granted 250GB. Registered users have the opportunity to apply for more storage. Users are strongly encouraged to use https://hicexplorer.usegalaxy.eu web server if high compute resources are required. Galaxy HiCExplorer is GDPR compliant; deleted datasets will be permanently removed within 14 days and the data of unregistered users is deleted after an inactivity of 90 days.

## TRAINING

To support researchers in their analysis of Hi-C, cHi-C or scHi-C data, tutorials and a detailed documentation are available on https://hicexplorer.readthedocs.io and https://schicexplorer.readthedocs.io. As presented in (29), the guided tours for novice users of Galaxy as well as the Galaxy HiCExplorer specific tutorial are available on the Galaxy Training Network (43). The cHi-C tutorial uses Promoter cHi-C example data to guide users through the complete analysis workflow starting from building a cHi-C contact matrix, creating a background model, detecting significant and differential interactions to a plotting of the viewpoints. The tutorial of the single-cell data explains the barcoding, the mapping, creation and merging of scHi-C matrices. Moreover it shows different clustering techniques including the dimension reduction and the visual representation of the clustering.

## DISCUSSION

The presented web server on https://hicexplorer.usegalaxy.eu gives researchers the opportunity to focus on their data analysis in a user friendly, reproducible and computationally powerful environment. With the deep integration of HiCExplorer into the Galaxy environment, users are now able to combine their Hi-C, cHi-C (Promoter cHi-C, HiChIP) or scHi-C data with their data from other high-throughput assays like ChIP-Seq or RNA-Seq and run multi-omics analyses, all within their web browser. Galaxy HiCExplorer is suited for both experts and newcomers to the Hi-C field, thanks to the provided tutorials that give all users a clear introduction on how to use HiCExplorer for their data analyses. Moreover, the tools recently added to HiCExplorer offer the possibility to resolve the dynamic chromatin topology inherent to different cell types provided by scHi-C. The automated management of a large number of cells in the scHi-C pipeline will help researchers to decipher the principles of chromatin folding in the context of cell cycle and cell type specificity. Moreover, the new tools of Galaxy HiCExplorer are able to analyse precise interactions between regulatory regions and their target genes assisted by cHi-C techniques. This expansion of Galaxy HiCExplorer allows for a better understanding of how 3D structure of a genome may affect an organism’s phenotype.

## Supplementary Material

gkaa220_Supplemental_File
