# Supplementary material for: Galaxy HiCExplorer 3: a web server for reproducible Hi-C, capture Hi-C and single-cell Hi-C data analysis, quality control and visualization
Source: Nucleic Acids Res. 2020 Apr 17;48(W1):W177–84. doi: 10.1093/nar/gkaa220 (PMC7319437; doi:10.1093/nar/gkaa220)
Supplement: gkaa220_Supplemental_File [file gkaa220_supplemental_file.pdf]

Supplementary material: Galaxy HiCExplorer 3: a web server for reproducible Hi-C, capture Hi-C and single-cell Hi-C data analysis, quality control and visualisation

1 HiCExplorer methods

1.1 hicNormalize

In the HiCExplorer software the term *normalize* is used in the context of adjusting the interaction values to the same value range. To achieve this, three methods are offered.
In the following the interaction matrix is defined as:

$$ICM = \begin{bmatrix} ic_{00} & \cdots & ic_{0n} \\ \vdots & \dots & \vdots \\ ic_{n0} & \cdots & ic_{nn} \end{bmatrix} \tag{1}$$

1.1.1 Smallest

This mode normalizes the read coverage of all given matrices ICM_l to the sum of the lowest read coverage present:

$$read\ coverage_l = \sum ICM_l \tag{2}$$

$$min\_index = argmin(read\ coverage) \tag{3}$$

$$adjust\_factor = \frac{\sum ICM_l}{\sum ICM_{min\_index}} \tag{4}$$

$$ic\_l_{j,k} = \frac{ic\_l_{j,k}}{adjust\_factor} \tag{5}$$

1.1.2 Norm range

The *norm range* mode normalizes each interaction matrix ICM independently to a 0 to 1 value range.

$$max\_value = \max{(ICM)} \tag{6}$$

$$min\_value = \min{(ICM)} \tag{7}$$

$$min\_max\_difference = max\_value - min\_value \tag{8}$$

$$ic_{i,j} = \frac{ic_{i,j} - min\_value}{min\_max\_difference} \tag{9}$$

1.1.3 Multiplicative mode

The *multiplicative mode* gives the option to multiply each interaction with a user defined value.

$$ic_{i,j} = ic_{i,j} * value \tag{10}$$

1.2 hicAverageRegions

hicAverageRegions takes as input a bed file with regions of interest. The user can define if the start, end or the center (end - start) should be considered as the reference point. Based on a reference point $icm_{i,j}$ and the user given range r, a sub-matrix per reference point is extracted:

$$ICM\_sub = \begin{bmatrix} ic_{i-r,j-r} & \cdots & ic_{i-r,j+r} \\ \vdots & icm_{i,j} & \vdots \\ ic_{i+r,j-r} & \cdots & ic_{i+r,j+r} \end{bmatrix} \quad (11)$$

All sub-matrices ICM_sub are added to one matrix and is divided by the number of sub-matrices. This resulting matrix is called the average region matrix.

1.3 hicPlotSVL

For each chromosome of each interaction matrix the short vs. long range distance ratio is computed as:

$$short\_range = \sum_{i=0}^{i<minRange} \sum_{j=0}^{j<minRange} ic_{i,j} \quad (12)$$

$$long\_range = \sum_{i=minRange}^{i<maxRange} \sum_{j=minRange}^{j<maxRange} ic_{i,j} \quad (13)$$

$$svl = \frac{short\_range}{long\_range} \quad (14)$$

All short vs long range values are ordered by the chromosomes and between different samples a Wilcoxon rank-sum is computed. The rank-sum test determines if two samples have a different ratio (small p-value) or not.

1.4 hicCompartmentalization

This tool helps in studying the polarization of the compartments by ordering the values of $PC1$ in an ascending manner and re-ordering their corresponding bins on the observed/expected matrix, we call it the 'polarization matrix'. With this method, all the bins with negative $PC1$ values (representative of inactive compartment (B)) should be shifted to the top/left corner of polarization matrix and all those with positive values (representative of active compartment (A)) should be moved at bottom/right cornet of the matrix. If there will be a clear compartmentalization on the given genome, it is expected that the sum of the contacts in these two corners be larger than the sum of the contacts on the two other corners of the matrix which contain the in-between compartments contacts.
The ascending ordering happens after dividing the values into a given number of quantiles, therefore the polarization matrix dimension is $quantiles * quantiles$. To make the polarization plot by counting the contacts of the polarization matrix, we apply the following method on each bin of the polarization matrix:

$$within\_comps = \sum matrix[0:b, 0:b] + \sum matrix[q-b:q, q-b:q];$$

$$between\_comps = \sum matrix[0:b, q-b:q] + \sum matrix[q-b:q, 0:b]; \quad (15)$$

$$within\_to\_between = \frac{within\_comps}{between\_comps}$$

Where b is the $bin + 1$ and q is the given number of quantiles.

2 Capture Hi-C

2.1 Background model

The user given reference point with the up- and downstream given distance is defined as the viewpoint. A relative distance is defined as the distance up- or downstream to a reference point.
To build the background model, all viewpoints from all samples are considered. Per relative distance rd over all viewpoints v one continuous negative binomial distribution is fitted:

$$X_{rd} \sim cNB_{rd}(r_{rd}, p_{rd}) \quad (16)$$

The continuous negative binomial distribution is created by exchanging the binomial coefficient of the probability mass function by gamma functions. Continuous negative binomial functions are used by edgeR [1, 2]; moreover, it was discussed on the website stackexchange[1] how to generalize negative binomial functions. This continuous negative binomial function is also used in the loop detection[2].

$$f(k, r, p) = \frac{\Gamma(k + r)}{\Gamma(k + 1) * \Gamma(r)} p^k (1 - p)^r \tag{17}$$

The p-value of an interaction i at the relative distance rd is given as:

$$pvalue\ of\ i = P(x \geq i) = 1 - \sum_{k=0}^{i-1} f_{rd}(k, r_{rd}, p_{rd}) \tag{18}$$

Additionally, the mean background per relative distance rd over all viewpoints v is computed.

2.2 Significant interaction detection

The detection of significant interactions is accomplished in three steps:

1. Loose p-value: all interactions which have this p-value or less are accepted as a candidate
2. x-Fold: all interactions with a interaction value $value * x - fold > mean\_background_{rd}$ are accepted as a candidate
3. For all interactions: if their neighbor interaction is a candidate too, consider their interaction as one and add them together. Add neighboring elements together as long they fulfil condition 1 or 3. Recompute all p-values for the new interaction and accept as significant if their p-value from cNB_{rd} is $p - value \leq threshold.$

2.3 Differential test

All interactions of interest are tested with Fisher's exact test or the chi^2 contingency test. Values for the test are always the interaction value of the reference point and the interaction value of the interaction of interest. These values are used to test against a second sample (e.g. wild type).

3 scHiCExplorer methods

scHiCExplorer uses traditional clustering algorithms which require a two dimensional matrix as an input but the nature of a Hi-C matrix is that it is already present in two dimensions; leading to three dimensions.
Let all single-cell Hi-C matrices ICM be given as $n \times n$ and each pixel as $icm_{k,l}$. Each Hi-C interaction matrix is flattened to one dimension and is stacked together with all the other flattened matrices to one two dimensional matrix $scICM$ where each row i resents therefore a cell, each feature j an interaction. An interaction at $scICM_{i,j}$ is equal to the single-cell Hi-C matrix of cell i and the interaction at position j is $j = (k * n) + l$. This results in the matrix $scICM$ with $i \times (n * n)$.

3.1 Dimension reduction

The raw clustering approaches of *scHicCluster* do not use any dimension reduction technique and operate directly on the matrix $scICM$. This can be problematic because the number of dimensions can go to the millions or even billions, depending on the resolution on the Hi-C matrices. Moreover, the clustering results are bad. Please consider Supplement Figure 1a, 1b and the cluster profile Supplement Figure 4a, 4b.

3.1.1 PCA

To compute the principle components, first the covariance matrix on $scICM$ is generated and then the eigenvectors are calculated on this matrix. Only the first i componets are considered, resulting in a dimension reduced matrix $scPCA$ of $i \times i$.

[1] https://stats.stackexchange.com/questions/310676/continuous-generalization-of-the-negative-binomial-distribution/311927
[2] https://www.biorxiv.org/content/early/2020/03/06/2020.03.05.979096

3.1.2 K-nearest neighbors

The k-nearest neighbors graph approach computes on $scICM$ for each cell i the i-nearest neighbors based on the euclidean distance. With this approach the dimensions can be reduced to $i \times i$ and each pixel i, j represents the euclidean distance between the two cells. However, the user can define a different value for the k-nn and is therefore able to reduce the compute time.

3.1.3 Approximate nearest neighbors: MinHash

The MinHash approach computes approximate nearest neighbors via an approximation of the Jaccard similarity. Moreover, it offers the option to precompute the Jaccard similarity and based on the subset of nearest neighbors the exact nearest neighbors via the euclidean distance can be computed. Please consider Wolff 2020: Approximate k-nearest neighbors graph for single-cell Hi-C dimensional reduction with MinHash[3] for more details.

3.1.4 Short vs long range ratio

For each single-cell Hi-C matrix ICM with $n \times n$ the short vs long range ration per chromosome is computed as described in Section 1.3. Let the number of all present single-cell matrices be i. All ratios per chromosome of all single-cell Hi-C matrices are stacked together resulting in a dimension reduced matrix $scSVL$ with $i \times |chromosomes|$ dimensions.

3.1.5 A/B compartments

For each single-cell Hi-C matrix ICM with $n \times n$ the A/B compartments are computed per chromosome and the first principal component is taken as the vector describing the matrix. Let the number of all present single-cell matrices be i. All first principal components of all single-cell Hi-C matrices are stacked together resulting in a dimension reduced matrix $scABC$ with $i \times n$ dimensions.

3.2 Clustering

As clustering methods k-means and spectral clustering are offered. Please consider the following Figures 1, 2, 3 and 4 for a comparison of the dimension reduction techniques and the quality of the clustering.

[3]http://dx.doi.org/10.1101/2020.03.05.978569

References

[1] Mark D Robinson, Davis J McCarthy, and Gordon K Smyth. edger: a bioconductor package for differential expression analysis of digital gene expression data. *Bioinformatics*, 26(1):139–140, 2010.

[2] Davis J McCarthy, Yunshun Chen, and Gordon K Smyth. Differential expression analysis of multifactor rna-seq experiments with respect to biological variation. *Nucleic acids research*, 40(10):4288–4297, 2012.

[3] Takashi Nagano, Yaniv Lubling, Csilla Várnai, Carmel Dudley, Wing Leung, Yael Baran, Netta Mendelson Cohen, Steven Wingett, Peter Fraser, and Amos Tanay. Cell-cycle dynamics of chromosomal organization at single-cell resolution. *Nature*, 547(7661):61, 2017.

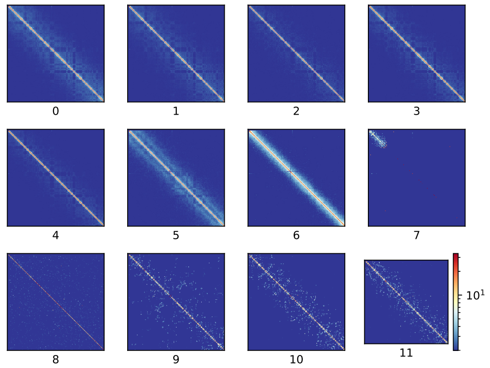

(a) Raw K-means

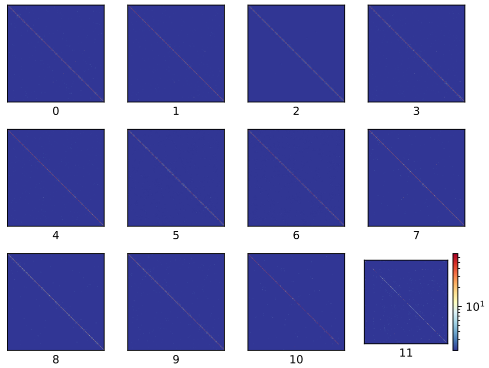

(b) Raw Spectral

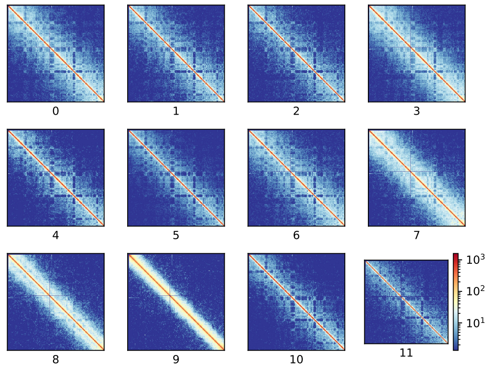

(c) Sklearn k-nn k = 100 K-means

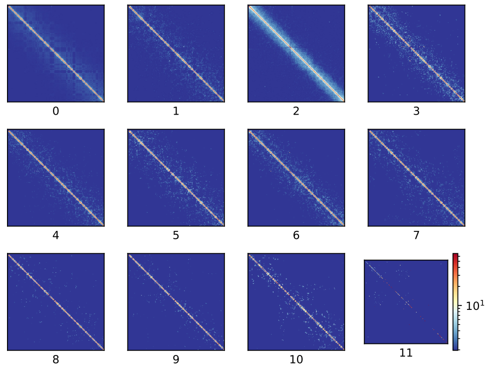

(d) Sklearn k-nn k = 100 Spectral

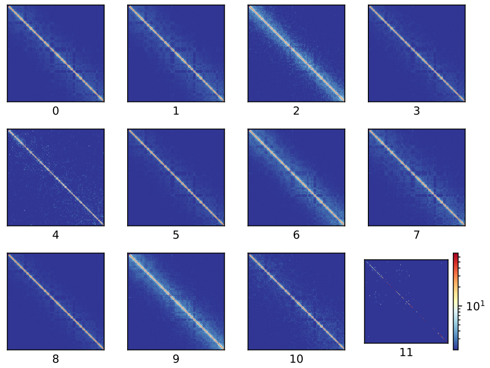

(e) Sklearn k-nn k = 2460 K-means

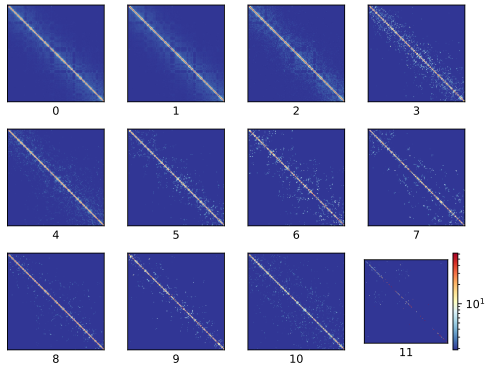

(f) Sklearn k-nn k = 2460 Spectral

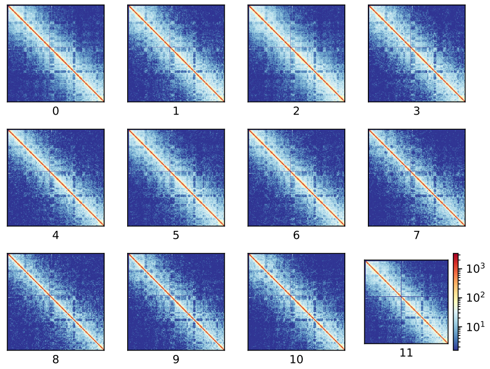

(g) Principal component analysis K-Means

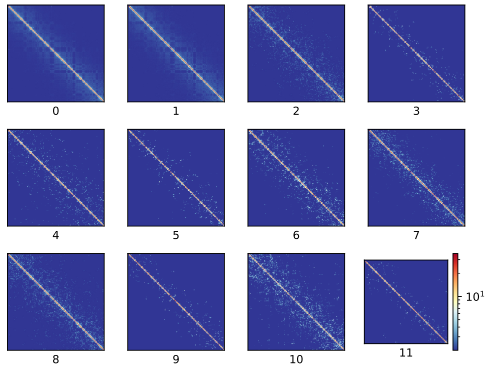

(h) Principal component analysis Spectral

Figure 1: Consensus matrices of the different clusters on 2460 cells from [3] Diploid cells, chromosome 1. K-Means and spectral clustering was used on the different dimension reduced scHi-C matrices. Results from scHicCluster on raw data (1a, 1b) and on dimension reduced data with *k-nearest neighbors* (1c, 1d, 1e, 1f) and *PCA* (1g, 1h).

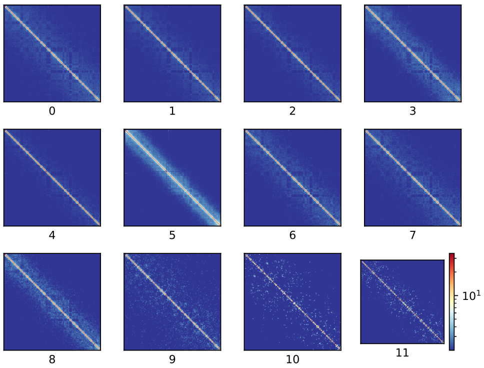

(a) MinHash k-nn k = 100 K-means

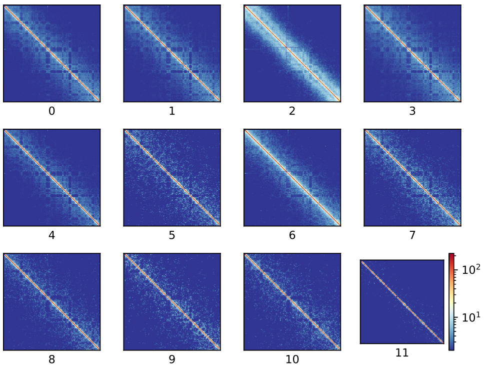

(b) MinHash k-nn k = 100 Spectral

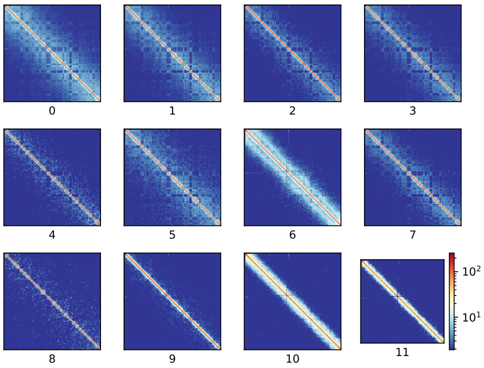

(c) MinHash k-nn k = 2460 K-means

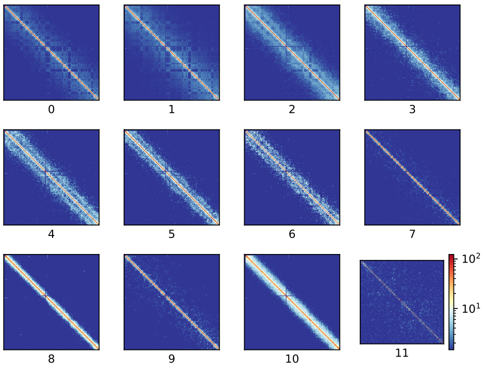

(d) MinHash k-nn k = 2460 Spectral

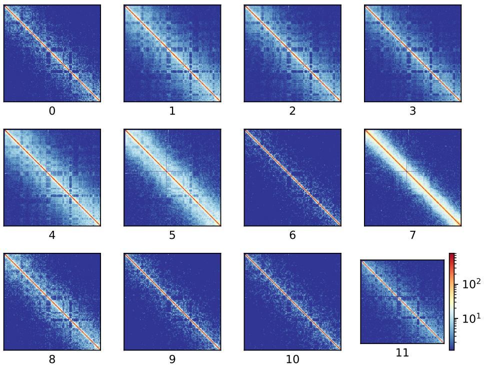

(e) MinHash exact mode k-nn k = 100 K-means

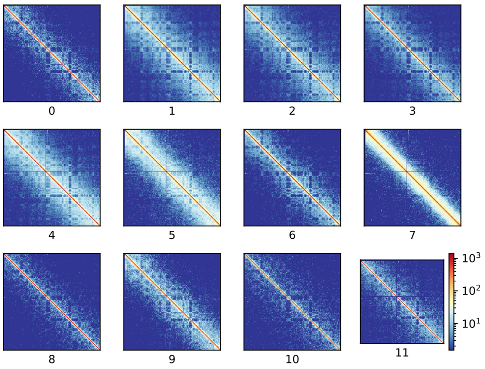

(f) MinHash exact mode k-nn k = 100 Spectral

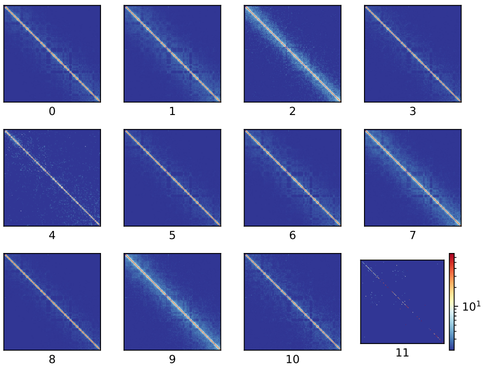

(g) MinHash exact mode k-nn k = 2460 K-means

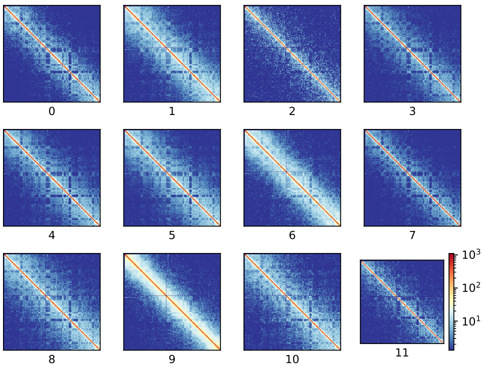

(h) MinHash exact mode k-nn k = 100 Spectral

Figure 2: Consensus matrices of the different clusters on 2460 cells from [3] Diploid cells, chromosome 1. K-Means and spectral clustering were applied on results from scHicClusterMinHash.

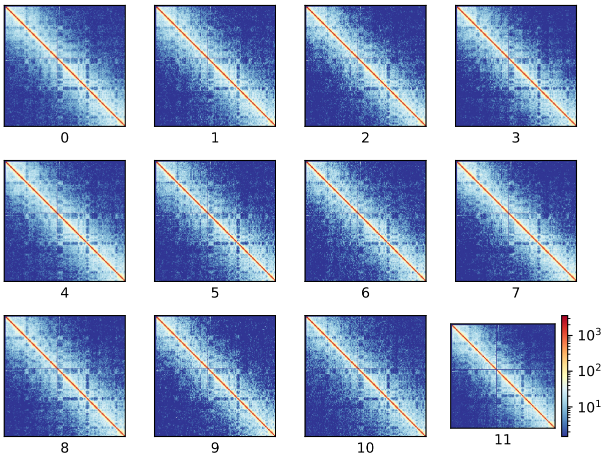

(a) A/B compartments K-Means

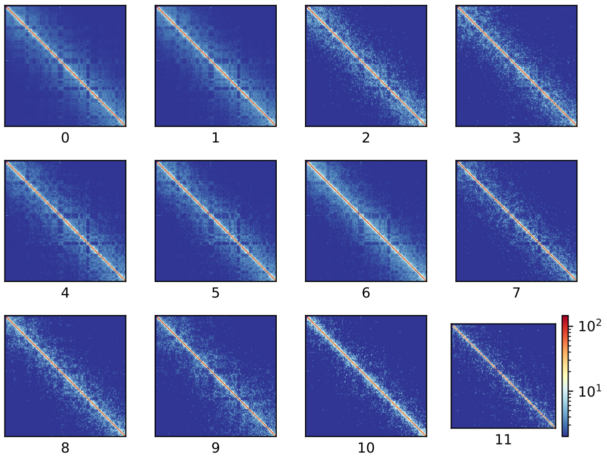

(b) A/B compartments Spectral

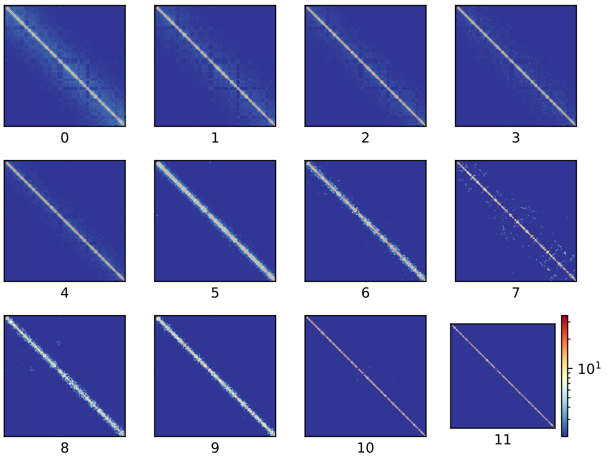

(c) SVL K-Means

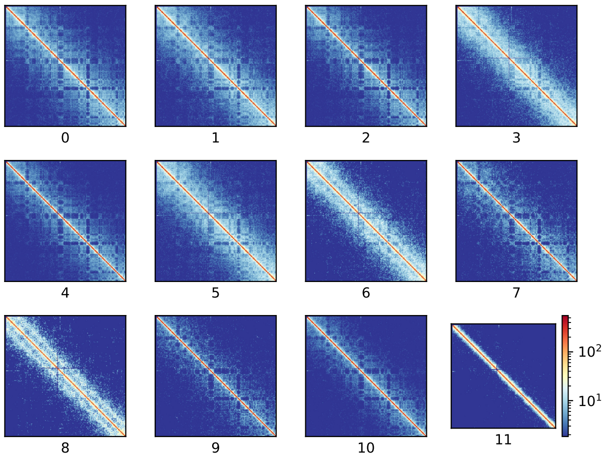

(d) SVL Spectral

Figure 3: Consensus matrices of the different clusters on 2460 cells from [3] Diploid cells, chromosome 1. K-Means and spectral clustering were applied on results from scHicClusterCompartments (3a, 3b) and scHicClusterSVL (3c, 3d).

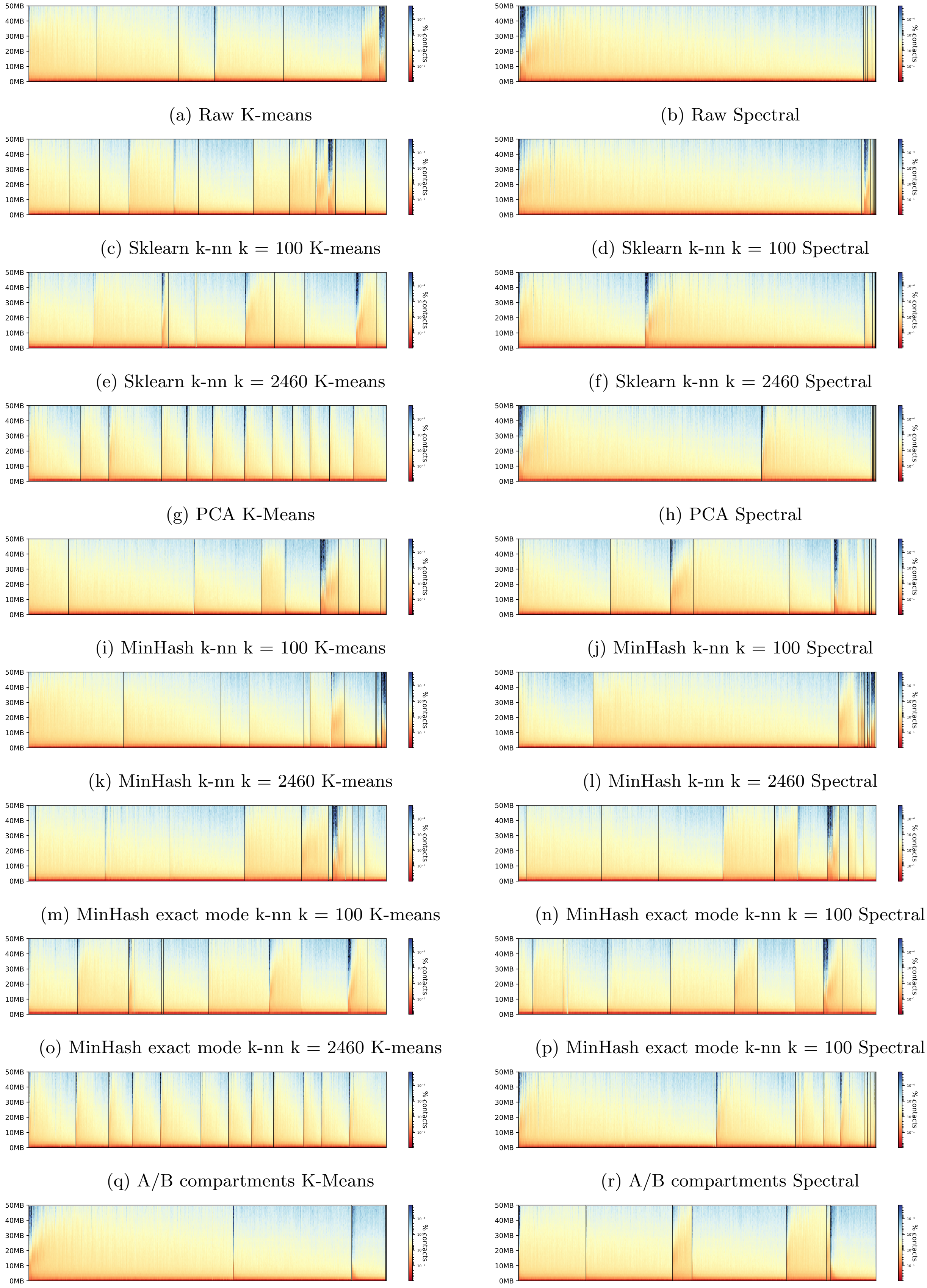

(a) Raw K-means

(b) Raw Spectral

(c) Sklearn k-nn k = 100 K-means

(d) Sklearn k-nn k = 100 Spectral

(e) Sklearn k-nn k = 2460 K-means

(f) Sklearn k-nn k = 2460 Spectral

(g) PCA K-Means

(h) PCA Spectral

(i) MinHash k-nn k = 100 K-means

(j) MinHash k-nn k = 100 Spectral

(k) MinHash k-nn k = 2460 K-means

(l) MinHash k-nn k = 2460 Spectral

(m) MinHash exact mode k-nn k = 100 K-means

(n) MinHash exact mode k-nn k = 100 Spectral

(o) MinHash exact mode k-nn k = 2460 K-means

(p) MinHash exact mode k-nn k = 100 Spectral

(q) A/B compartments K-Means

(r) A/B compartments Spectral

(s) SVL K-Means

(t) SVL Spectral

Figure 4: Cluster profile of the different clusters on 2460 cells from [3] Diploid cells. K-Means and spectral clustering were applied on the different dimension reduced scHi-C matrices. Results from scHicCluster on raw (4a, 4b) with knn (4c, 4d, 4e, 4f) mode, PCA (4g, 4h); scHicClusterMinHash (4i, 4j, 4k, 4l, 4m, 4n, 4o, 4p); scHicClusterCompartments (4q, 4r) and scHicClusterSVL (4s, 4t).

Method	Runtime	Memory
Raw and K-Means	1:52 h	33 GB
Raw and Spectral	3:02 min	6.7 GB
PCA and K-Means	7:27 min	220 GB
PCA and Spectral	7:39 min	220 GB
sklearn k-nn k = 100 and K-means	2:11 min	6.7 GB
sklearn k-nn k = 100 and Spectral	3:40 min	6.7 GB
sklearn k-nn k = 2460 and K-means	18:41 min	6.7 GB
sklearn k-nn k = 2460 and Spectral	3:44 min	6.7 GB
MinHash k = 100 and K-means	2:18 min	6.7 GB
MinHash k = 100 and Spectral	3:27 min	6.7 GB
MinHash k = 2460 and K-means	6:59 min	6.7 GB
MinHash k = 2460 and Spectral	3:20 min	6.7 GB
MinHash exact mode k = 100 and K-means	4:25 min	6.7 GB
MinHash exact mode k = 100 and Spectral	2:56 min	6.7 GB
MinHash exact mode k = 2460 and K-means	1:12 h	6.7 GB
MinHash exact mode k = 2460 and Spectral	1:03 h	6.7 GB
A/B compartments K-means	40:52 min	6.7 GB
A/B compartments Spectral	1:10 h h	6.7 GB
SVL K-means	1:51 min h	6.7 GB
SVL compartments Spectral	1:52 min h	6.7 GB

(a) Data: 1 MB resolution, with 2460 cells.

Method	Runtime	Memory
Raw and K-Means	-	> 1 TB
Raw and Spectral	-	> 1 TB
PCA and K-Means	-	> 1 TB
PCA and Spectral	-	> 1 TB
sklearn k-NN and K-Means	-	> 1 TB
sklearn k-NN and Spectral	-	> 1 TB
MinHash k = 2508 and K-Means	1:13 h	53 GB
MinHash k = 2508 and Spectral	1:04 h	53 GB
MinHash exact mode k = 2508 and K-Means	3:19 h	53 GB
MinHash exact mode k = 2508 and Spectral	3:11 h	53 GB
MinHash exact mode k = 50 and K-Mans	1:08 h	53 GB
MinHash exact mode k = 50 and Spectral	1:03 h	53 GB
MinHash exact mode k = 200 and K-Mans	1:17 h	53 GB
MinHash exact mode k = 200 and Spectral	1:09 h	53 GB
A/B compartments K-means	> 14 days	- GB
A/B compartments Spectral	> 14 days	- GB
SVL K-means	1:11 h	6.7 GB
SVL compartments Spectral	1:14 h	6.7 GB

(b) Data: 10 kb resolution, with 2460 cells. The raw matrix, PCA and sklearn k-nn methods requested more than the available 1 TB of memory and could not be computed; A/B compartments were computing for 14 days and the computation has been canceled by us.

Table 1: Data from [3] Diploid cells, with 12 clusters. For clustering K-means and spectral clustering were used, MinHash with 800 hash functions. All results computed on 2x XEON E5-2630 v4 @ 2.20GHz 2x 10 cores / 2x 20 threads, 1 TB memory.
